# Modeling Pharmaceutical Batch Cooling Crystallization Processes Using Computational Fluid Dynamics Coupled with a One-Dimensional Population Balance Model

**DOI:** 10.1021/acs.cgd.5c00980

**Published:** 2026-01-16

**Authors:** Diana M. Camacho Corzo, Juliet A. Figueroa Rosette, Abdul Samad Rana, Cai Y. Ma, Kevin J. Roberts, Tariq Mahmud

**Affiliations:** Centre for Digital Design of Drug Products, School of Chemical and Process Engineering, The University of Leeds, Leeds LS2 9JT, U.K.

## Abstract

The batch cooling crystallization of the α polymorphic form of l-glutamic acid from aqueous solution in a kilo-scale 20 L pharmaceutical batch crystallizer is simulated using a multiphase computational fluid dynamics (CFD) model coupled with a one-dimensional population balance equation (PBE). The predicted three-dimensional spatial and temporal distributions of turbulent kinetic energy, supersaturation, nucleation rate, and solid volume fraction provide a high fidelity and very detailed insights into the interplay between crystallizer hydrodynamics and crystallization process kinetics and their resultant impact upon the resulting crystal size distributions (CSDs). Comparison of the CFD-PBE modeling results with published experimental data (Liang, 2002) demonstrates the model’s predictive capability by reproducing the measured final CSDs with an acceptable degree of accuracy. An increase in impeller speed is found to increase both the measured and predicted CSD curves shift toward smaller particles sizes. In terms of the spatial variations of process parameters, the evolution of CSD during the crystallization process reveals significant variation of the evolving CSD at the early stages (between 45 and 40 °C) of the crystallization process, which is relatively invariant in the later stages (between 30 and 20 °C), consistent with the reduction of solution supersaturation within the batch process. The simulation results under different agitation rates reveal that at the higher rates, smaller crystals are produced due to a greater level of turbulence and higher supersaturation at an early stage of the process. Detailed sensitivity analysis on the effect of crystallization kinetics on the predicted CSD emphasizes the need for using reliable kinetic data relevant to the crystallization conditions being simulated.

## Graphical abstract

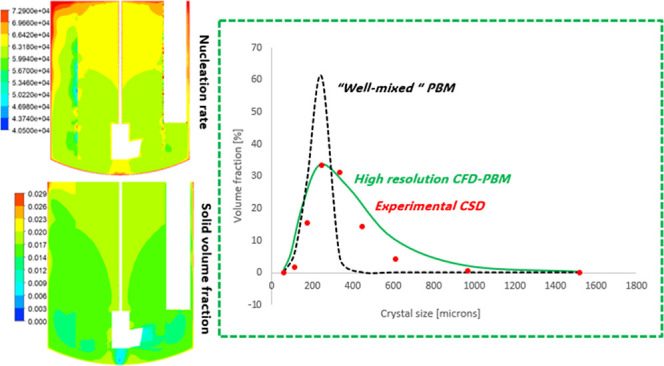

## 1 Introduction

### 1.1 Background

Batch cooling crystallization is one of the most common crystallization processes used for the isolation and purification of intermediate and final solid products in the pharmaceutical industry. It is also commonly used in a wide range of chemical processes throughout the industrial sector, for example, agrochemicals and fine/specialty chemicals. The design and operating conditions of crystallizers can directly influence the physical/chemical properties of the final active pharmaceutical ingredients (APIs), such as the CSD, crystal shape (morphology), polymorphic form, purity, and product yield. These attributes can determine, in turn, the quality and the performance of the ingredients and their resultant formulation. The crystal size and shape can also influence the performance of the downstream post-crystallization filtration and drying operations as well as the performance of the unit operations used in secondary manufacturing. In the pharmaceutical industry, crystallization process development at a laboratory scale and its subsequent scaling up for manufacturing is largely carried out via experimental trial and error methods. Such a time-consuming and materials intensive approach lacks environmental sustainability, adversely impacting R&D costs and prolonging product times to market. In contrast, adoption of a digital twin approach^1^ using first-principles-based modeling tools can significantly reduce the need for experiments at different scales, providing an opportunity for faster regulatory approval and shorter product time to market, consistent with a significant reduction in R&D costs.

In a previous publication,^2^ a state-of-the-art CFD-based modeling framework for the digital design and optimization of crystallization processes was proposed. This approach encompasses CFD for modeling crystallizer’s hydrodynamics, coupling a one-dimensional (1D) population balance model (PBM) with a multiphase CFD for the prediction of three-dimensional (3D) distributions of crystallization process parameters and CSD, and finally a multizonal process model informed via CFD and incorporating a morphological-PBM (e.g., refs 3 and 4 ). The latter would be based on the crystal facet growth rates and their kinetics obtained experimentally via machine learning coupled with a high-resolution digital microscopy^5,6^ for the prediction of crystal size and shape distributions. In the previous paper,^2^ an assessment of CFD methodologies for the predictions of hydrodynamics and macroparameters, such as power number, impeller flow number, and secondary circulation flow number, in a typical pharmaceutical crystallizer has been reported. In the wake of this study, the present work is concerned with the further development of the modeling strategy focusing on the development and assessment of a CFD-PBM methodology for reliable predictions of process parameters and the final product CSD as a function of crystallizer operating conditions.

In stirred tank crystallizers, from laboratory through to industrial scale sizes, highly inhomogeneous and transient hydrodynamics and mixing conditions can exist, resulting in nonuniform distributions of crystallization process parameters within the crystallizer such as temperature and solute concentration resulting in variation in solution supersaturation (defined as *C*/C* with *C* being the solute concentration and C* being the equilibrium concentration at the same temperature). The interrelationship between the hydrodynamics/mixing and the distributions of these parameters is highly complex and poorly understood. Generally, lumped-parameter mechanistic models encompassing solution of a 1D population balance equation (PBE), based on the well-mixed assumption, tend to be used for the prediction of CSD.^7–9^ Although such models can be convenient and useful for an initial assessment of the crystallizer performance, neglecting local variations of hydrodynamic and process parameters can lead to incorrect estimation of the overall nucleation and crystal growth rates, which can result in the evolution of a CSD which could fail the desired product specification. There is, therefore, a need for a more rigorous distributed-parameter modeling approach based on a multiphase CFD model coupled with a PBE in order to capture the effect of nonuniform distributions of these parameters on the predicted crystal properties.

### 1.2 Previous Modeling Studies

Previous work has coupled CFD with 1D-PBE and has applied different approaches for the modeling of crystallization processes in stirred tank crystallizers (see reviews in refs 10–12 ). However, many of these have focused on rapid precipitation processes (e.g., refs 13–20 ), antisolvent^21–23^ and evaporative^24^ crystallization processes, as well as on continuous crystallization using jet^25^ or oscillatory baffled crystallizers.^26^ In contrast, fully coupled CFD-PBM simulations of batch cooling crystallization processes have been quite limited,^27–31^ despite the extensive use of these processes in API and fine chemical manufacturing. Some studies have also carried out a hybrid CFD-compartmental modeling approach for batch cooling crystallization whereby the crystallizer was subdivided into a number of interconnected well-mixed compartments based on the CFD-predicted flow field data.^32–35^ Previous CFD-PBM simulations have often been carried out in vessels of standard configurations, typically with four symmetrical baffles and agitation using a Rushton turbine, neither of which is commonly used industrially.

Three different numerical methods have mainly been applied to solve the 1D-PBE (see the review in ref 36). The most commonly used approach has utilized the method of moments^37,38^ using both standard and quadrature methods of moments. In this, the PBE has been expressed as equations of moments in order to determine gross properties of the particle population distribution, for example, total number, length, area, and volume of particles per unit volume of mixture. Although this approach has been found to be both computationally efficient and convenient to integrate with CFD, information about CSD is not readily available. Hence, the CSD needs to be reconstructed from the moments by assuming a size distribution function (for details, see ref 36). A robust and more accurate method of solving the PBE is the discrete method,^39,40^ which has been employed in some studies (e.g., refs 20, 22, 28, and 29 ). In this, the CSD is divided into several discrete size classes or bins, and the PBE is converted into discretized equations using, for example, finite difference methods (for details of discretization schemes, see ref 36). While this approach has the advantage that it can provide the CSD directly, the number of bins must be defined a priori, and a large number of bins may be required for modeling a wider particle size range.^20,41^ The discretization methods employed in this approach have been found to be computationally intensive when compared to the methods of moments. However, they are recommended for processes where a detailed shape of the CSD is required and where the physical and mechanical properties of crystals are strongly depended on the CSD.^19^

Previous CFD-PBM simulations of crystallization processes in stirred tank crystallizers also generally have assumed single-phase or pseudohomogeneous flow where the particles are very small and hence assumed to follow the liquid-phase streamlines. This would be unrealistic for cooling crystallization processes where crystal sizes can be quite large. Finally, flow turbulence within the crystallizer has been usually modeled using the eddy-viscosity-based turbulence models, such as the standard *k*-*ε* model or its variants, which can be deficient in capturing accurately the mean and turbulence flow fields in agitated vessels.^42,43^

### 1.3 Present Contributions

This study distinguishes itself from previous work by employing a comprehensive crystallization process modeling methodology based on a three-dimensional (3D), transient Eulerian–Eulerian two-phase CFD which is based on the kinetic theory of granular flow. In addition, the most advanced turbulence model within the RANS (Reynolds-averaged Navier–Stokes) modeling framework has been used, which has been fully coupled with a 1D-PBE solved via a discrete method and applied to an industry-relevant crystallizer.^44^ This overall approach, as outlined in Figure 1, enables a direct quantification of the interplay between local hydrodynamics, mixing, and the kinetics of the crystallization process (nucleation and crystal growth) throughout the vessel.

**Figure 1:**
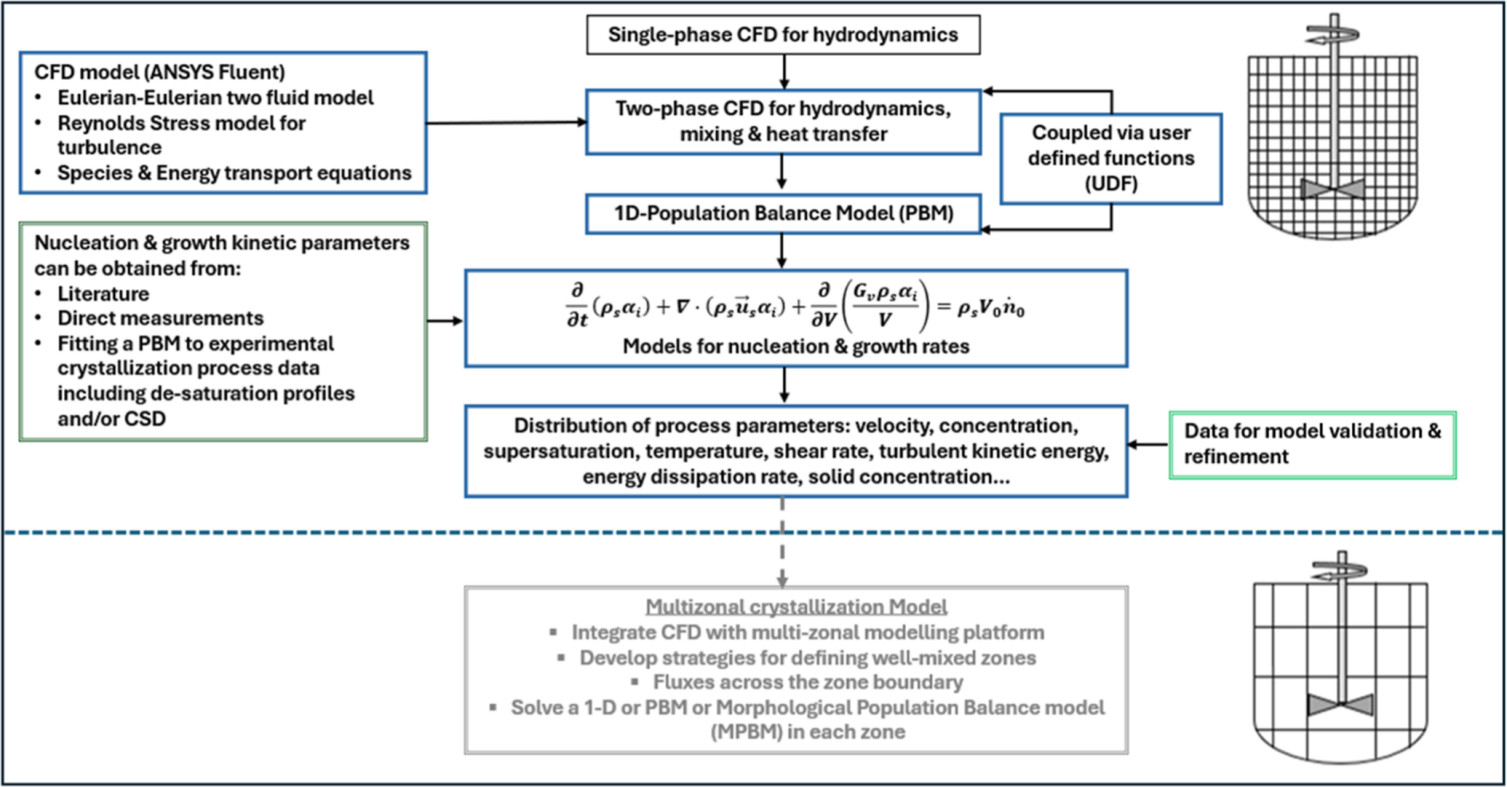
Overview of the coupled CFD-PBM crystallization process modeling framework.

The present study represents one component of a broader modeling strategy, which allows the level of coupling between the CFD and PBM to be tailored according to the desired balance between model fidelity and computational demand. While the current work focuses on the fully coupled CFD-PBM implementation, the same framework can readily be extended to a reduced-order representation in which the crystallizer is discretized into a series of interconnected well-mixed zones where the PBE is solved within each zone. This zonal approach,^45,46^ although not applied herein, provides a computationally efficient alternative for modeling large-scale crystallizers.

The present study is based on the batch cooling study of the crystallization of the α-form of l-glutamic acid (α-LGA) from an aqueous solution as described by Liang,^44^ which used a representative kilo-scale 20 L glass-jacketed pharmaceutical crystallizer equipped with a single cylindrical baffle and agitated by a retreat curve impeller (RCI). LGA was selected because it has two well-defined polymorphic forms, in common with many pharmaceutical compounds, which yields different crystal morphologies: the metastable, prismatic α-form and the stable, needle like β-form.^47^ The processing conditions for the crystallization of α-LGA^44^ is well known and defined. In principle, a morphologically based PBM (e.g., refs 3 and 4 ) could be used to predict the evolution of crystal size and shape during crystallization processes for LGA, but the corresponding computational time involved can be substantial.^48^ However, the volume-equivalent crystal size used in the 1D-PBM can better represent the prismatic α-form than the needle-like β-form, albeit losing some of the shape information. Hence, α-LGA was selected for this study. In this, the nucleation and crystal growth kinetics were represented by power-law models using model constants obtained from both literature^49^ and in-house measurements.^50^ The overall aim of the coupled CFD-PBM predictions was to provide a comprehensive insight into the crystallization process via examination of the spatial and temporal distributions of hydrodynamic parameters, temperature, solute concentration, supersaturation, and solid concentration within a representative batch crystallization process.

## 2 Crystallization Modeling Methodology

### 2.1 Description of the Experimental Process

The modeling work draws down on the experimental studies carried out by Liang.^44^ Figure 2 shows a schematic of the 20 L dish-bottom crystallizer used in this work with details of the vessel geometry, baffle, and impeller dimensions being given in Table 1. In this study, the batch cooling crystallization experiments were carried out at a solution cooling rate of 0.6 °C/min using a solution of 99% pure LGA in distilled water with an initial concentration of 43 g of LGA/1000 g of water (corresponding to a solution saturation temperature of 70 °C) at different impeller speeds: 100, 150, 200, and 250 rpm. In-process measurements of the final volume-based CSD were carried out using ultrasonic attenuation spectrometry (USS) using a flow-through cell coupled to the USS system.^51,52^ The USS technique has been well described elsewhere.^51,52^ The conditions of the experiments simulated in this study are given in Table 2.

**Figure 2:**
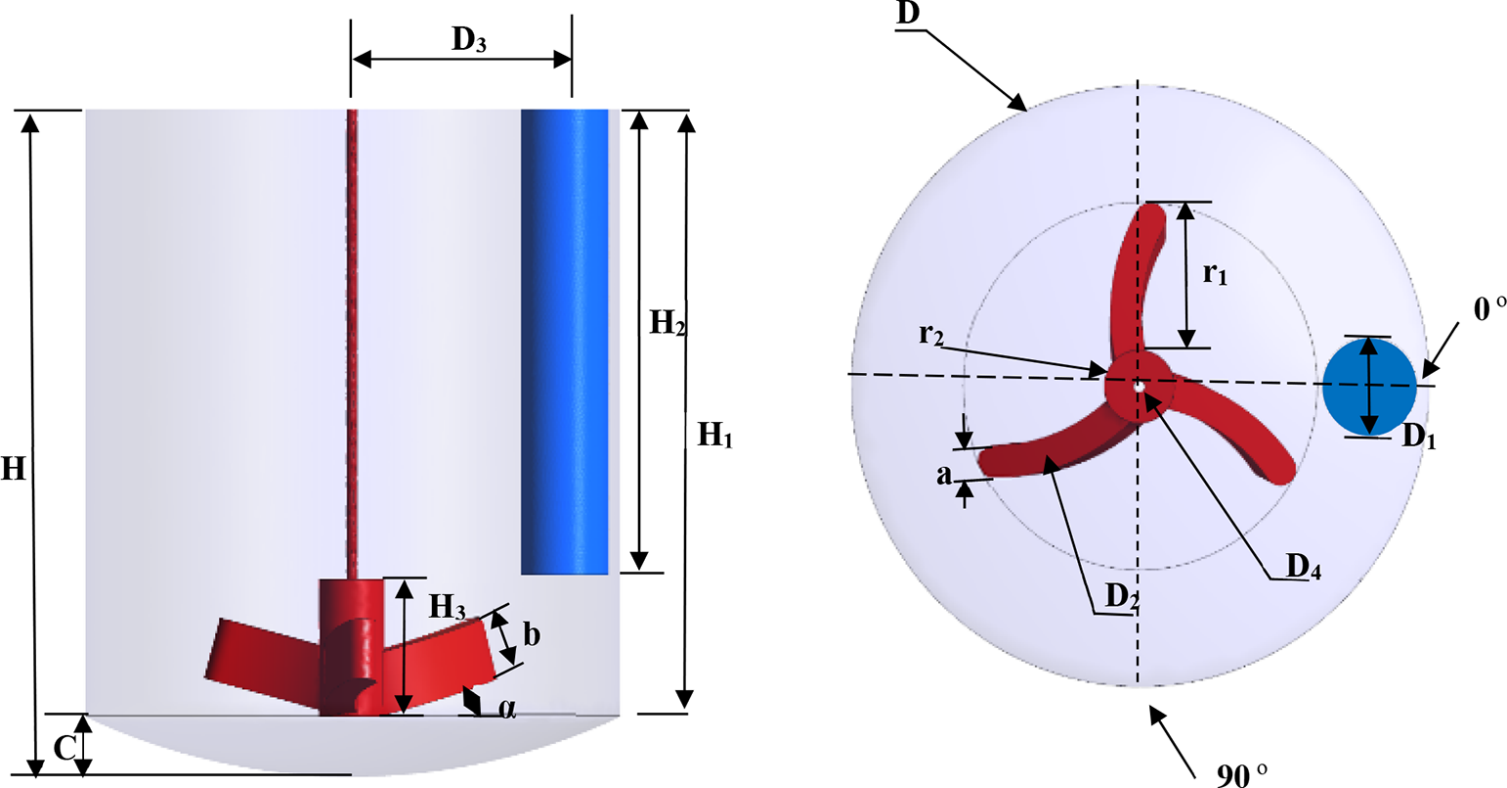
Configuration of the stirred tank crystallizer with a RCI and a cylindrical baffle used by Liang.^44^

**Table 1:** Crystallizer Geometry and Impeller Dimensions [in mm]

*H*	294	*H*_1_	261	*H*_2_	183
*H*_3_	75	*D*	294	*D*_1_	48
*D*_2_	36	*D*_3_	120	*D*_4_	6
*r*_1_	90	*r*_2_	86	*α*	15°
*a*	16	*b*	34	*C*	33

**Table 2:** Conditions of Crystallization Experiments Simulated in this Study

Stirrer speed *N* [rpm]	Reynolds number $\frac{Nd^{2}\rho }{\mu }$	Cooling rate [°C/min]	Solute concentration [g/L]	Temperature range for cooling [°C]	
				initial	final
100	5.27 × 10^4^	0.6	45	75	20
150	7.91 × 10^4^				

### 2.2 CFD Modeling Approach

The CFD-PBM methodology used in this study is illustrated in Figure 1. A Eulerian–Eulerian multiphase CFD approach was used to model the slurry flow field. Although a free-surface vortex can form in a single baffled agitated vessel, it is expected that the viscosity of the slurry would be high enough to suppress the vortex depth considerably for the agitation rates simulated in this study. As revealed in our previous hydrodynamic simulations in the same crystallizer^2^ for water/glycerol mixtures, the vortex depth decreased by 37% when the viscosity increased from 0.0037 to 0.0108 cP. Furthermore, the predicted vortex depths at the impeller speeds of 100 and 150 rpm were found to be quite small in that study, leading us to assume a flat liquid surface in the present study. Although the volume-of-fluid (VOF) method was successfully integrated with the single-phase CFD for capturing vortex formation in this crystallizer in the Stage-1 work,^2^ for a three-phase fluid system consisting of a binary liquid mixture of water and dissolved LGA with varying composition, solid particles and air in the ullage region of the crystallizer proved notoriously difficult and challenging using commercial CFD codes such as ANSYS Fluent. Our initial attempts using the multiphase methodology resulted in physically unrealistic air–liquid interface profiles. Further efforts to revise the methodology in order to generate the correct predictions are ongoing.

#### 2.2.1 Governing Equations for Two-Phase Flow

A multifluid model based on the kinetic theory of granular flow accounting for the particle–particle interaction is used to describe the flow fields of the solid–liquid mixture in the crystallizer. The general instantaneous mass and momentum conservation equations for a transient two-phase flow in the Eulerian–Eulerian framework are given below.^41^ The liquid phase (an aqueous solution of LGA) is considered as the primary phase, and the solid phase (LGA crystals) is considered as the secondary phase.

##### 2.2.1.1 Liquid-Phase Equations

The mass conservation equation takes the form:

1 $$\frac{\partial }{\partial t}\left(\alpha_{l}\rho_{l}\right)+\nabla \cdot \left(\alpha_{l}\rho_{l}{\mathrm{u⃗}}_{l}\right)=0$$

where *α*_l_ is the volume fraction, *ρ*_l_ is the density, and *u⃗*_l_ is the velocity vector of the liquid phase.

The momentum conservation equation is given by

2 $$\frac{\partial }{\partial t}\left(\alpha_{l}\rho_{l}{\mathrm{u⃗}}_{l}\right)+\nabla \cdot \left(\alpha_{l}\rho_{l}{\mathrm{u⃗}}_{l}{\mathrm{u⃗}}_{l}\right)=-\alpha_{l}\nabla p+\nabla \cdot {\mathrm{\tau ̿}}_{l}+\alpha_{l}\rho_{l}\mathrm{g⃗}+K_{sl}\left({\mathrm{u⃗}}_{s}-{\mathrm{u⃗}}_{l}\right)+{\mathrm{F⃗}}_{l}$$

where *p* is the pressure sheared by both phases, ${\mathrm{\tau ̿}}_{l}$ is the liquid-phase stress–strain tensor, ***F⃗***_i_ is the external body force arising from the centrifugal and Coriolis forces, *g⃗* is the gravitational acceleration, *K*_sl_ is the interphase momentum exchange coefficient described by eq 6, and *u⃗*_s_ is the velocity vector of the solid phase. The stress–strain tensor is defined as

3 $${\mathrm{\tau ̿}}_{l}=\alpha_{l}\mu_{l}\left(\nabla {\mathrm{u⃗}}_{l}+\nabla {{\mathrm{u⃗}}_{l}}^{T}\right)+\alpha_{l}\left(\lambda_{l}-\frac{2}{3}\mu_{l}\right)\nabla \cdot {\mathrm{u⃗}}_{l}\mathrm{I̿}$$

where *μ*_l_ and *λ*_l_ are the shear and bulk viscosity, respectively, and I̿ is the unit tensor.

##### 2.2.1.2 Solid-Phase Equations

The mass conservation equation is given by

4 $$\frac{\partial }{\partial t}\left(\alpha_{s}\rho_{s}\right)+\nabla \cdot \left(\alpha_{s}\rho_{s}{\mathrm{u⃗}}_{s}\right)=0$$

where *α*_s_ is the volume fraction and *ρ*_s_ is the density of the solid phase. The sum of liquid- and solid-phase volume fractions is equal to unity: *α*_l_ + *α*_s_ = 1.

The momentum conservation equation is given by

5 $$\frac{\partial }{\partial t}\left(\alpha_{s}\rho_{s}{\mathrm{u⃗}}_{s}\right)+\nabla \cdot \left(\alpha_{s}\rho_{s}{\mathrm{u⃗}}_{s}{\mathrm{u⃗}}_{s}\right)=-\alpha_{s}\nabla p-\nabla p_{s}+\nabla \cdot {\mathrm{\tau ̿}}_{s}+\alpha_{s}\rho_{s}\mathrm{g⃗}+K_{ls}\left({\mathrm{u⃗}}_{l}-{\mathrm{u⃗}}_{s}\right)+{\mathrm{F⃗}}_{s}$$

where *p*_s_ is the solids pressure representing the normal stress due to the particle–particle interactions, ${\mathrm{\tau ̿}}_{s}$ is the solids stress tensor, and other terms have their usual meaning as defined for eq 2. In the present simulations, the lift and virtual mass forces are neglected as the effect of these forces on the predicted flow fields in agitated vessels was found negligible in previous studies (e.g., refs 53–55 ). In common with previous studies (e.g., refs 53, 56, and 57 ), the turbulent dispersion force accounting for the interphase turbulent momentum transfer is not included.

The coupling between the liquid and solid phases is achieved via the interphase momentum exchange coefficient, *K*_sl_ (= *K*_ls_), which is defined as

6 $$K_{sl}=\frac{3}{4}\frac{C_{D}}{d_{s}}\alpha_{s}\rho_{l}\left|{\mathrm{u⃗}}_{s}-{\mathrm{u⃗}}_{l}\right|$$

where *C*_D_ is the drag coefficient and *d*_s_ is the particle diameter. *C*_D_ is obtained from the drag model of Schiller and Naumann,^58^ which has been used in a number of modeling studies of solid–liquid flow as well as crystallization in agitated vessels (e.g., refs 30, 55, 56, 59, and 60 ), and is given by

7 $$C_{D}=\{\begin{array}{ll} 24\left(1+0.15{{Re}_{s}}^{0.687}\right)/{Re}_{s}, & {Re}_{s}\leq 1000\\ 0.44, & {Re}_{s}>1000 \end{array}$$

where Re_s_ is the particle Reynolds number defined based on the relative velocity between the two phases as

8 $${Re}_{s}=\frac{\rho_{l}\left|{\mathrm{u⃗}}_{s}-{\mathrm{u⃗}}_{l}\right|d_{s}}{\mu_{l}}$$

The solid-phase stress tensor ( ${\mathrm{\tau ̿}}_{s}$ ) in eq 5 is expressed as

9 $${\mathrm{\tau ̿}}_{s}=\alpha_{s}\mu_{s}\left(\nabla {\mathrm{u⃗}}_{s}+\nabla {{\mathrm{u⃗}}_{s}}^{T}\right)+\alpha_{s}\left(\lambda_{s}-\frac{2}{3}\mu_{s}\right)\nabla \cdot {\mathrm{u⃗}}_{s}\mathrm{I̿}$$

where *μ*_s_ is the solid shear viscosity and *λ*_s_ is the bulk viscosity. The viscosities in eq 9 and solids pressure in eq 5 are obtained from their respective constitutive equations derived from the kinetic theory of granular flow in terms of the granular temperature (*Θ*_s_), which is proportional to the kinetic energy associated with the random motions of the particles. The conservation equation for granular temperature^41^ (defined as $\frac{1}{3}{u_{s}^{\prime }}^{2}$ , where *u*_s_^′^ is the fluctuating solids velocity) is given by

$$\frac{3}{2}\left[\frac{\partial }{\partial t}\left(\alpha_{s}\rho_{s}\Theta_{s}\right)+\nabla \cdot \left(\alpha_{s}\rho_{s}{\mathrm{u⃗}}_{s}\Theta_{s}\right)\right]=-\left(p_{s}\mathrm{I̿}+{\mathrm{\tau ̿}}_{s}\right):\nabla {\mathrm{u⃗}}_{s}+\nabla \cdot \left(k_{\Theta_{s}}\nabla \Theta_{s}\right)-\gamma_{s}\;+\phi_{\;ls}$$ 10

where $-\left(p_{s}\mathrm{I̿}+{\mathrm{\tau ̿}}_{s}\right):\nabla {\mathrm{u⃗}}_{s}$ is the generation of energy by the solid stress tensor, *k*_Θs_∇*Θ*_s_ is the diffusion of energy, *γ*_s_ is the rate of energy dissipation due to collisions between particles, and *ϕ*_ls_ (= –3*K*_ls_ *Θ*_s_) is the transfer of kinetic energy from the solid to the liquid phase. The granular temperature was obtained by solving an algebraic formulation of eq 10 where the convection and diffusion terms are neglected.

##### 2.2.1.3 Turbulence Modeling

The RANS approach used in the simulation requires an appropriate turbulence closure to model the Reynolds stress tensor ( ${\mathrm{u⃗}}_{q}^{\prime }⊗{\mathrm{u⃗}}_{q}^{\prime }$ ) resulting from the time averaging of momentum conservation equations. In general, the modeling of turbulence in multiphase flows is more challenging compared to that in single-phase flows due to the additional complexity arising from the interactions between the continuous and the dispersed phase turbulence, and this requires reliable turbulence models.^55,60^ The eddy-viscosity based two-equation turbulence models, such as the standard *k*-*ε* model and its variants, have commonly been employed for the simulation of solid–liquid flow in agitated vessels (see the review in Shi and Rzehak^55^ and the references therein), as well as for the simulation of crystallization processes (see references cited in Section 1.2). It is well established that such turbulence models cannot adequately capture the underlying hydrodynamic characteristics, particularly in the impeller region, where strong anisotropy prevails. Our previous modeling study^2^ of single-phase flow in the same crystallizer using the Shear stress transport (SST) and Reynolds stress transport (RST) models of turbulence has revealed improved performance of the latter model.

Three types of turbulence modeling approaches can be used for multiphase flows, namely, the mixture, the dispersed, and the phasic (or per phase) model. It should be noted that the phasic RST model is not available in the ANSYS Fluent. However, comparisons of these approaches^54,61,62^ using the *k*–*ε* turbulence model have revealed that for low solid loadings in agitated vessels, the performances of all three approaches are similar. In the simulations presented here, an RST mixture turbulence model has been used. In this approach, it is assumed that both phases share the same turbulence field, and the differential transport equations for individual components of Reynolds stresses in terms of mixture properties and mixture velocities are solved. The transport equations for Reynolds stresses can be expressed in a general form as^60^

11 $$\frac{\partial }{\partial t}\left(\rho_{m}R_{ij}\right)+\frac{\partial }{\partial x_{k}}\left(\rho_{m}{\mathrm{u⃗}}_{m}R_{ij}\right)=\frac{\partial }{\partial x_{k}}\left[\mu_{m}\frac{\partial }{\partial x_{k}}\left(R_{ij}\right)\right]+P_{ij}+G_{ij}+\phi_{ij}-\rho_{m}\varepsilon_{ij}$$

where *ρ*_m_ and *μ*_m_ are the mixture density and viscosity, respectively, *u⃗*_m_ is the mixture velocity, *R*_ij_ is the Reynolds stresses, *P*_ij_ is the stress production term, *G*_ij_ is an additional production term due to the system rotation, *ϕ*_ij_ is the pressure–strain redistribution term, which was modeled using the linear pressure–strain model of Launder et al.^63^ following Camacho Corzo et al.,^2^ and *ε*_ij_ is the viscous dissipation rate of turbulent kinetic energy, which is obtained by solving its transport equation.

#### 2.2.2 Scalar Conservation Equations

The spatial and temporal distributions of dissolved LGA concentration in the solution are obtained by solving a species conservation equation expressed as

12 $$\frac{\partial }{\partial t}\left(\alpha_{l}\rho_{l}Y_{LGA}\right)+\nabla \cdot \left(\alpha_{l}\rho_{l}{\mathrm{u⃗}}_{l}Y_{LGA}\right)=-\nabla \cdot \alpha_{l}\left(\rho_{l}D_{m}+\frac{\mu_{t}}{{Sc}_{t}}\right)\nabla Y_{LGA}+S_{LGA}$$

where *Y*_LGA_ is the mass fraction of LGA, *D*_m_ is the molecular diffusion coefficient, Sc_t_ (= *μ*_t_/*ρ*_l_ *D*_t_) is the turbulent Schmidt number, *D*_t_ is the turbulent diffusivity, *μ*_t_ is the turbulent viscosity, and *S*_LGA_ is the rate of consumption of LGA due to nucleation and crystal growth.

The temperature distributions in the liquid and solid phases are determined by solving the energy conservation equation given by

13 $$\frac{\partial }{\partial t}\left(\alpha_{q}\rho_{q}h_{q}\right)+\nabla \cdot \left(\alpha_{q}\rho_{q}{\mathrm{u⃗}}_{q}h_{q}\right)=-\nabla \cdot \left({\vec{q}}_{q}+{\vec{q}}_{q}^{t}\right)+Q_{pq}$$

where *h*_q_ is the specific enthalpy of the *q* ^th^ phase, ${\vec{q}}_{q}$ is the conductive heat flux, ${\vec{q}}_{q}^{t}$ is the turbulent heat flux, and *Q*_pq_ (= –*Q*_qp_) is the volumetric rate of convective heat transfer between the two phases. It should be noted that the heat input from the impeller, viscous dissipation, and enthalpy of crystallization are not included in the conservation equation. The turbulent heat flux is modeled as

14 $${\vec{q}}_{q}^{t}=-k_{t}\nabla T$$

where *k*_t_ is the turbulent thermal conductivity, which can be expressed in terms of the turbulent Prandtl number, Pr_t_, as *k*_t_ = *μ_t_c*_p_/Pr_t_.

#### 2.2.3 Population Balance Equation

The 1D-PBE, appropriate for solving using the discrete method, is given by eq 15 (ANSYS Fluent 12.0 Population Balance Module Manual). In this method, the PBE is written for each discrete particle size class (or bin) *i* in terms of its volume fraction

15 $$\frac{\partial }{\partial t}\left(\rho_{s}\alpha_{i}\right)+\nabla \cdot \left(\rho_{s}{\mathrm{u⃗}}_{s}\alpha_{i}\right)+\frac{\partial }{\partial V}\left(\frac{G_{v}\rho_{s}\alpha_{i}}{V}\right)=\rho_{s}V_{0}{ṅ}_{0}$$

where α*_i_* (= *N*_i_ *V*_i_) is the volume fraction of particle size *i*, *V*_i_ is the volume of particle size *i*, and *N*_i_ denotes the number density of particles in size *i* given by

16 $$N_{i}\left(t\right)=\int_{V_{i}}^{V_{i+1}}n\left(V,t\right)\;dV$$

A solution variable defined by eq 17 is introduced

17 $$f_{i}=\frac{\alpha_{i}}{\alpha }$$

where *α* is the total volume fraction of the secondary phase.

In eq 15, the nucleation rate, *ṅ*_0_, represents the generation of particles of the smallest size *V*_0_ and *G*_v_ represents the volume-based crystal growth rate, which is discretized as follows:

18 $$\frac{\partial }{\partial V}\left(\frac{G_{v}\rho_{s}\alpha_{i}}{V}\right)=\rho_{s}V_{i}\left[\left(\frac{G_{v,i-1}N_{i-1}}{V_{i}-V_{i-1}}\right)-\left(\frac{G_{v,i}N_{i}}{V_{i+1}-V_{i}}\right)\right]$$

and the volumetric coordinate is discretized as

19 $$\frac{V_{i+1}}{V_{i}}=2^{q}$$

where *q* (= 1,2···) is designated as the ratio factor.

The birth and death terms in the PBE due to breakage and agglomeration are not included because these phenomena were not significant in the crystallization of α-LGA as observed in the images of crystals.^44^ The PBE is coupled with the secondary phase momentum conservation equation (eq 5) via the Sauter mean diameter, where *d*_32_ representing the particle size distribution defined as

20 $$d_{32}=\frac{\sum n_{i}d_{s,i}^{3}}{\sum n_{i}d_{s,i}^{2}}$$

where *n*_i_ and *d*_s__,__i_ are the number and diameter, respectively, of particles of size *i*.

#### 2.2.4 Crystallization Kinetics Models

The nucleation and crystal growth rates are expressed as a function of the solution relative (or absolute) supersaturation using the power-law models as follows:

21 $${ṅ}_{0}=k_{N}{\left(\sigma \right)}^{n}$$

22 $$G=k_{G}{\left(\sigma \right)}^{g}$$

where *k*_N_ and *k*_G_ are the rate constants, *n* and *g* are the exponents of the nucleation and growth rate models, respectively, and *σ* is the relative supersaturation expressed as *σ* = *S* – 1, where *S* is the supersaturation ratio defined as

23 $$S=\frac{C}{C^{*}}$$

where *C* and C* are the solute and the equilibrium saturation concentration, respectively, in mole of LGA/mol of solution. The solubility of α-LGA in water is given by^64^

24 $$C^{*}=0.08131-0.000595783T+1.1025810^{-6}T^{2}$$

where *T* is the solution temperature in degree Celsius.

The equations for the nucleation and crystal growth rates, and solubility are included in the PBE in ANSYS Fluent through User Defined Functions (UDF’s). The values of the parameters of the nucleation rate equation (eq 21) are obtained from Tai and Shei,^49^ while those in the crystal growth rate equation (eq 22) are also obtained from Tai and Shei^49^ as well as determined from the experimental data reported by Penchev.^50^

Tai and Shei^49^ carried out experiments in a 6 L vessel agitated by a four-blade pitch turbine at 600 rpm, where the LGA solution was crashed cooled from 10 °C above the saturation temperature to different selected crystallization temperatures. After equilibration, a slurry sample was withdrawn, and the CSD was determined using a laser diffraction particle size analyzer. A PBE (with no breakage or agglomeration terms) was solved to estimate the parameters of the nucleation and crystal growth rate expressions by fitting the experimental population density data. The values of the rate constant and exponent in the nucleation rate equation were found to be 4.02 × 10^6^ #/m^3^ s and 1.87, respectively, and for the crystal growth rate equation 9.76 × 10^–8^ m/s and 2.34, respectively, whereas Penchev^50^ performed experiments in a 20 L vessel agitated by a RCI at 100 rpm. The LGA solution saturated at 48 °C was cooled at linear rates of 0.1 and 0.2 °C/min from 60 to 10 °C. Measurements of CSD were performed via USS during the cooling of the solution. The reported crystal growth rate data as a function of relative supersaturation for both cooling rates are plotted in the present study in order to estimate the growth kinetics parameters. The fitting of the data using a power law, as depicted in Figure 3, provided the values of the rate constant and exponent of eq 22 as 2.80 × 10^–7^ m/s and 1.43, respectively.

**Figure 3:**
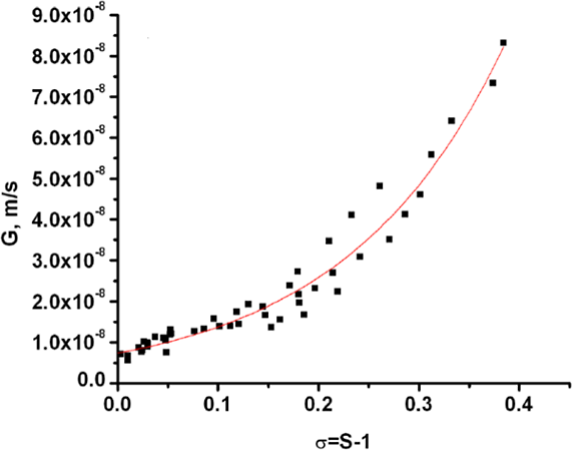
A plot of the crystal growth rate data (■) of Penchev^50^ as a function of relative supersaturation at cooling rates of 0.1 and 0.2 °C/min and a stirrer speed of 100 rpm for the estimation of growth kinetics parameters (red —best fit line).

## 3 Application of CFD-PBM for the Simulations of Experimental Cases

### 3.1 Computational Domain and Mesh

The 3D transient simulations were carried out using the sliding-mesh technique. The computational domain representing the experimental crystallizer^44^ (Figure 2) was discretized using an unstructured mesh consisting of 6 × 10^5^ tetrahedral cells with local refinements along the solid surfaces to resolve the boundary layer accurately. Figure 4a shows the computational mesh used in the simulations. The computational domain was divided into two regions, as illustrated in Figure 4b: the inner region encompassing the rotating impeller and the outer region containing the stationary baffle and vessel walls. Further details of the mesh generation and mesh independence study can be found in Camacho Corzo et al.^2^

**Figure 4:**
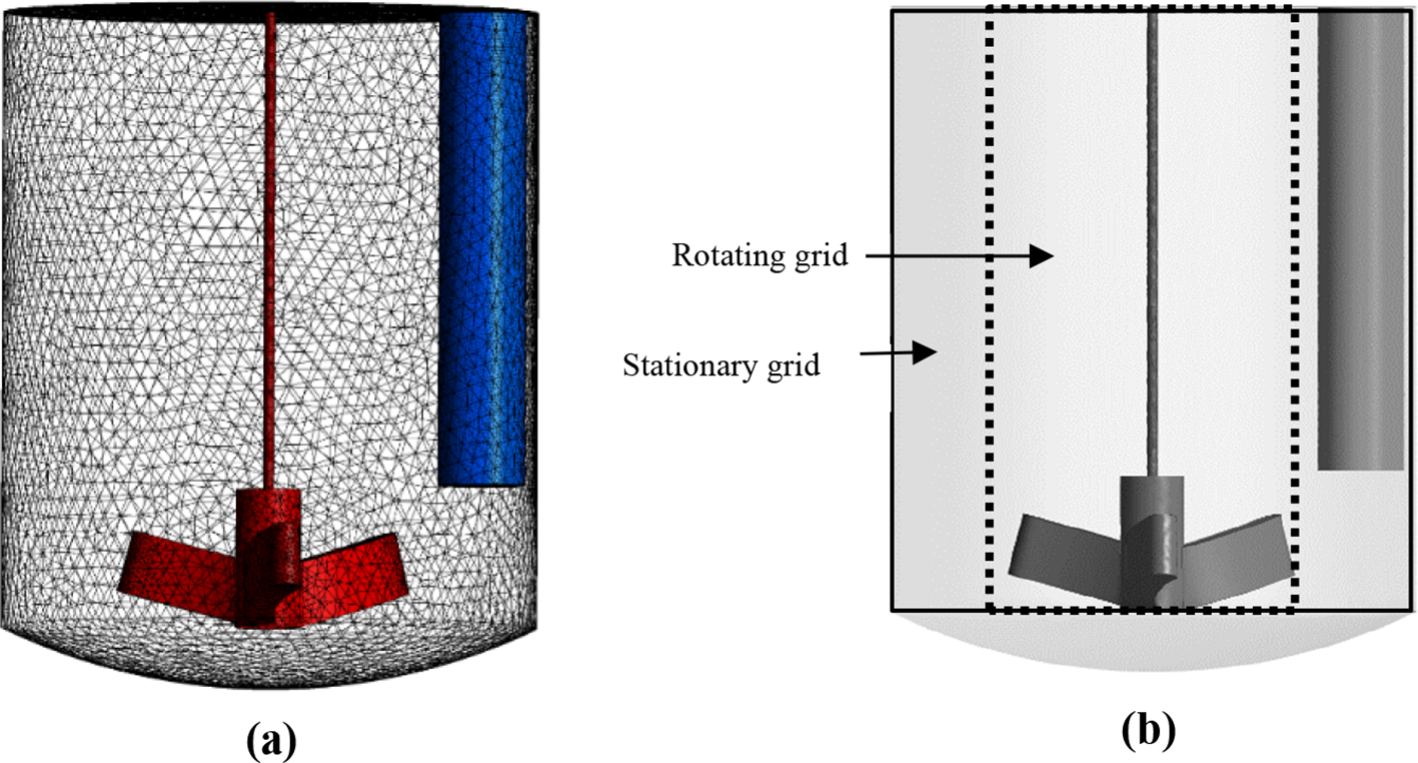
Crystallizer geometry and computational mesh (a) and representation of the computational domain using a sliding mesh technique (b).

### 3.2 Boundary Conditions and Solution Methods

Initially, the crystallizer contains a binary liquid mixture of dissolved LGA in water with a solute mass fraction of 0.043 corresponding to a saturation temperature of 70 °C. The no-slip boundary condition with appropriate wall functions was applied to all the vessel walls in contact with the liquid. A zero-shear boundary condition was applied at the top of the liquid surface. A constant heat flux of 2499 W/m^2^ corresponding to a cooling rate of 0.6 °C/min was applied as the thermal boundary condition to the side and bottom walls of the crystallizer.

A Multiple Reference Frame approach^41^ was used to generate initial values of the single-phase flow field. These results were used as the initial values to carry out transient two-phase flow simulations using the sliding mesh technique to model the rotating impeller and stationary baffle. A second-order upwind spatial discretization scheme was used for the convection terms in the governing equations in order to reduce the numerical diffusion errors. The transient terms were discretized using the first-order implicit method. A pressure-based solver using the SIMPLE algorithm^41^ was employed to solve the discretized continuity and momentum equations together with the boundary conditions for the velocity and pressure fields in order to ensure stability and convergence using the ANSYS Fluent-V17.1 CFD code. Target residuals were set to 1 × 10^–5^ with 20 iterations per time step, which was sufficient to achieve this target. A very small time step of 1 × 10^–8^ s was necessary initially to ensure solution stability, and as the solution approached toward convergence, the time step was gradually increased to 0.1 s. The simulations were run on an Intel­(R) Xeon­(R) CPU E5-278W v4 workstation @ 3.00 GHz (two processors) with 128 GB memory under the Windows 2012 operating system. The total computation time was approximately 6 weeks for the simulation of 1.2 h of the process time.

### 3.3 Simulation Cases

Four simulations were performed in order to evaluate the effect of variations in the crystallizer impeller speed and the crystallization kinetic parameters on the final product CSD. Simulation conditions are given in Table 3. Simulation Runs 1 and 3 examined the effect of the impeller speeds of 100 and 150 rpm, respectively, using the nucleation and crystal growth kinetics parameters of Tai and Shei^49^ for a cooling range of 70–20 °C. Runs 2 and 4 were performed to assess the crystal growth kinetics using the parameters obtained from Penchev^50^ for 100 and 150 rpm, respectively.

**Table 3:** CFD-PBM Simulation Conditions and Crystallization Kinetics Parameters

Run	Impeller speed (rpm)	Nucleation rate			Growth rate		
		*k*_N_	*n*	Reference	*k*_G_	*g*	Reference
1	100	4.02 × 10^6^	1.87	Tai and Shei^49^	9.76 × 10^–8^	2.34	Tai and Shei^49^
2	100				2.80 × 10^–7^	1.43	Penchev^50^
3	150				9.76 × 10^–8^	2.34	Tai and Shei^49^
4	150				2.80 × 10^–7^	1.43	Penchev^50^

## 4 Results and Discussion

### 4.1 Predicted Flow Fields

The predicted liquid flow fields for 100 rpm impeller speed (Run 1 in Table 3) on a vertical plane at the 0–180° angular position (see Figure 2) are depicted in Figure 5 in the form of velocity vectors. Figure 5a illustrates the initial liquid mixture flow field at 67 °C before the onset of crystallization, and Figure 5b is the flow field in the presence of crystals at the end of the crystallization at 20 °C. In both cases, some common features are evident between these flow fields, notably, downward flow along the impeller shaft toward the blade tip, as well as an upward flow near the vessel wall. The predictions also reveal that recirculation zones have been established in the top part of the vessel near the wall as well as under the cylindrical baffle and below the impeller. The maximum liquid-phase velocity was found to be 1.2 m/s (Figure 5a), which decreases slightly to 1.18 m/s (Figure 5b) as the concentration of the solid increases. Also, the maximum velocity of the liquid phase (1.18 m/s) was found to be slightly higher than that of the solid phase (1.13 m/s) on a plane at 90°, which suggests that the liquid flow is not significantly affected by the presence of a small amount of crystals having a maximum volume fraction of approximately 0.03. A recent study by Mousavi et al.^20^ using a CFD-PBM approach has reported that the multiphase flow field around the impeller is similar to that of a single-phase flow, especially for low solid concentration and small crystal sizes (10–50 μm). It should be noted that the drag coefficient and the level of turbulent fluctuations predicted by different drag laws and turbulence models, respectively, can significantly contribute to the difference between the velocity distributions of the two phases.^54,65^

**Figure 5:**
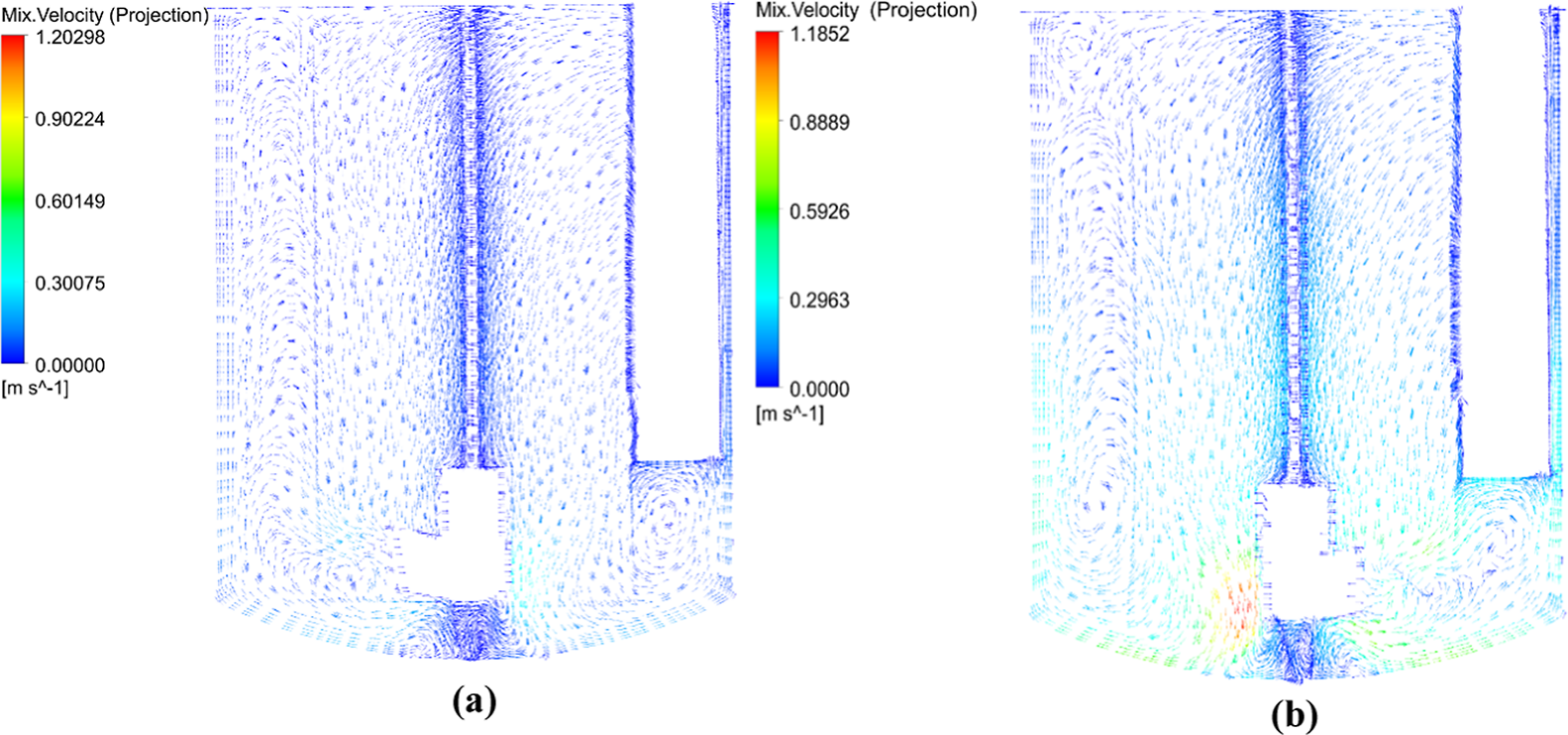
Predicted flow patterns for an impeller speed of 100 rpm (Run 1 in Table 3) on the 0–180° plane through the cylindrical baffle (a) at 67 °C (before the onset of crystallization) and (b) at 20 °C (end of the crystallization run).

### 4.2 Predicted Crystallization Process

Figure 6 illustrates the predicted global evolution of the crystallization process during cooling from 70 to 20 °C for Runs 1 and 3 at 100 and 150 rpm, respectively. The process parameters presented in Figure 6 have been averaged over the whole crystallizer volume. As can be seen, the LGA concentration starts to decrease at a temperature of around 45 °C for both the agitation rates, indicating the onset of crystallization. This would correspond to a metastable zone width of about 25 °C, which is broadly in line with previous measurements in 0.5 and 4.5 L crystallizers reported by Borissova et al.^47^ and Liang et al.,^66^ respectively. The supersaturation was found to initially increase as the temperature decreased to 45 °C, when the highest supersaturation levels of 1.52 and 1.53 for 100 and 150 rpm, respectively, were achieved, after 42 min into the process. Beyond this point, desupersaturation was found to occur as the solute concentration decreases due to nucleation and growth of crystals produced. The solute concentration reached a level very close to the equilibrium concentration (i.e., the solubility curve) after 65 min (30 °C). From this point onward, a small residual level of supersaturation of approximately 1.07, which is relatively constant, is generated by further cooling.

**Figure 6:**
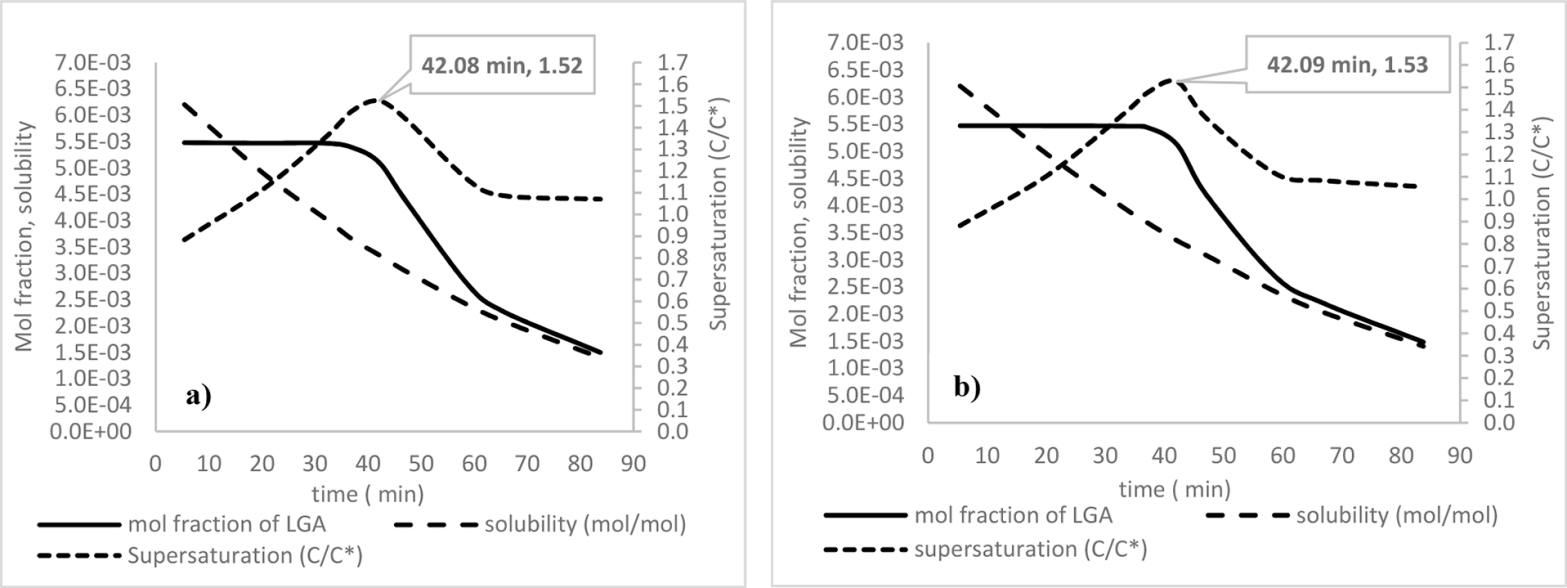
Predicted volume-averaged LGA concentration, supersaturation (*S* = *C*/C*), and the solubility curve at (a) 100 and (b) 150 rpm for cooling crystallization of LGA from 70 to 20 °C.

Figure 7 illustrates the predicted evolution of CSD at different temperatures during the crystallization process at an impeller speed of 100 rpm (Run 1). Examination of this data reveals that at the beginning of the process, a significant variation in the CSD is observed between 45 and 40 °C, which becomes relatively invariant during the final stage of the process between 30 and 20 °C, consistent with the low level of relatively constant supersaturation of 1.07 as shown in Figure 6a. The increase in crystal size due to crystal growth is manifested by the shift in the peak of the distribution toward the larger size values.

**Figure 7:**
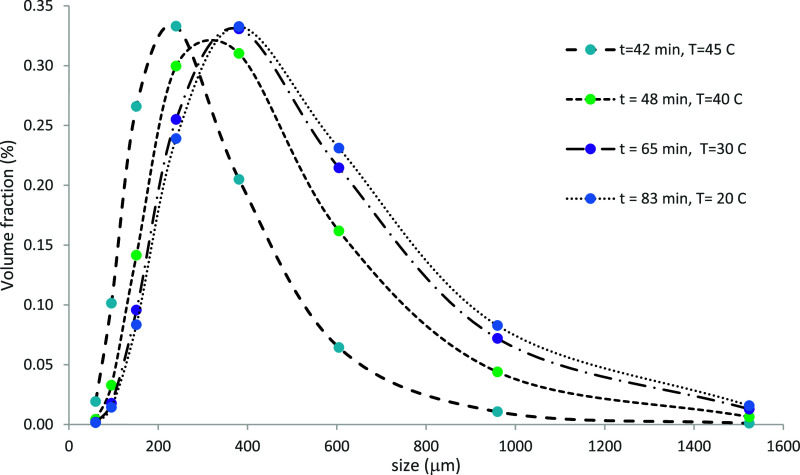
Predicted volume-averaged CSDs at different temperatures during the crystallization process at 100 rpm (Run 1).

### 4.3 Prediction of the Spatial Distributions of the Processing Environment

The spatial and temporal distributions of turbulent kinetic energy, nucleation rate, supersaturation, and solid volume fraction at different stages of the process at 100 and 150 rpm are illustrated in Figures 8 and 9, respectively. Supersaturation is the driving force for nucleation and crystal growth, and the level of supersaturation depends on the balance between the rate of consumption of solute due to crystallization and the rate of supersaturation generation via cooling. The values of supersaturation in the crystallizer depend on the local solute concentration and solution temperature, which determines the equilibrium concentration. It is worth noting that in a batch stirred tank crystallizer of a given configuration, the solute (and thereby supersaturation) and solid concentration distributions are also affected by the progress of mixing with time, which in turn depends on the bulk flow (i.e., convection) and turbulent fluctuating velocities (or eddy diffusion). As can be seen at the onset of crystallization (at 42 min, 45 °C), the dominant region of particle formation, as revealed by high nucleation rates, is located in the upper region of the crystallizer where high levels of supersaturation exist, resulting in extensive nucleation. High levels of supersaturation and nucleation rate are also noticeable along the crystallizer walls where heat transfer to the cooling jacket is highest and hence the solution temperature is at its lowest level. As nucleation is strongly promoted by high supersaturation, smaller crystals would be located in these regions where this process dominates over crystal growth. As the crystallization progresses, the solution starts to desupersaturate due to the consumption of solute by nucleation and crystal growth, and the supersaturation field can be observed to approach toward a more uniform distribution due to the progression of mixing with time. The maximum, as well as the volume-averaged supersaturation level, at the onset of crystallization increases slightly as the impeller speed is increased from 100 rpm (Figure 8) to 150 rpm (Figure 9). However, the region of maximum supersaturation near the top liquid surface shrinks significantly, and a more uniform distribution prevails over a much larger volume of the crystallizer because of the enhanced mixing at 150 rpm. The contours of nucleation rate, which reflect the supersaturation distributions, reveal that as the impeller speed increases, so does the maximum value of nucleation rate. Furthermore, higher values of nucleation rates are observed over the entire volume of the crystallizer.

**Figure 8:**
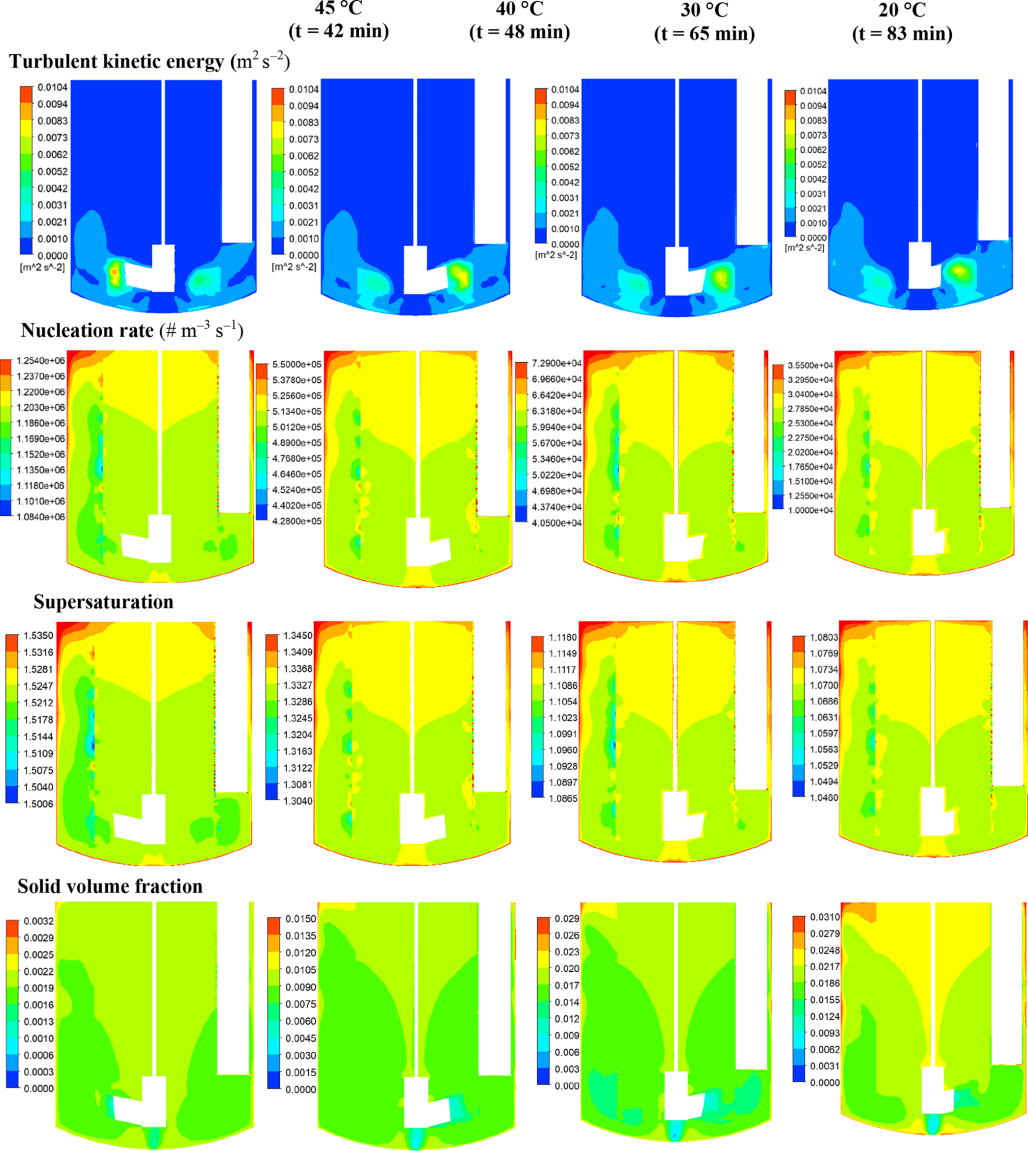
Predicted distributions of crystallization process parameters on the 0–180° vertical plane at 45 °C, 40 °C, 30 °C, and 20 °C for 100 rpm impeller speed: (a) turbulent kinetic energy, (b) nucleation rate, (c) supersaturation, and (d) solid volume fraction.

**Figure 9:**
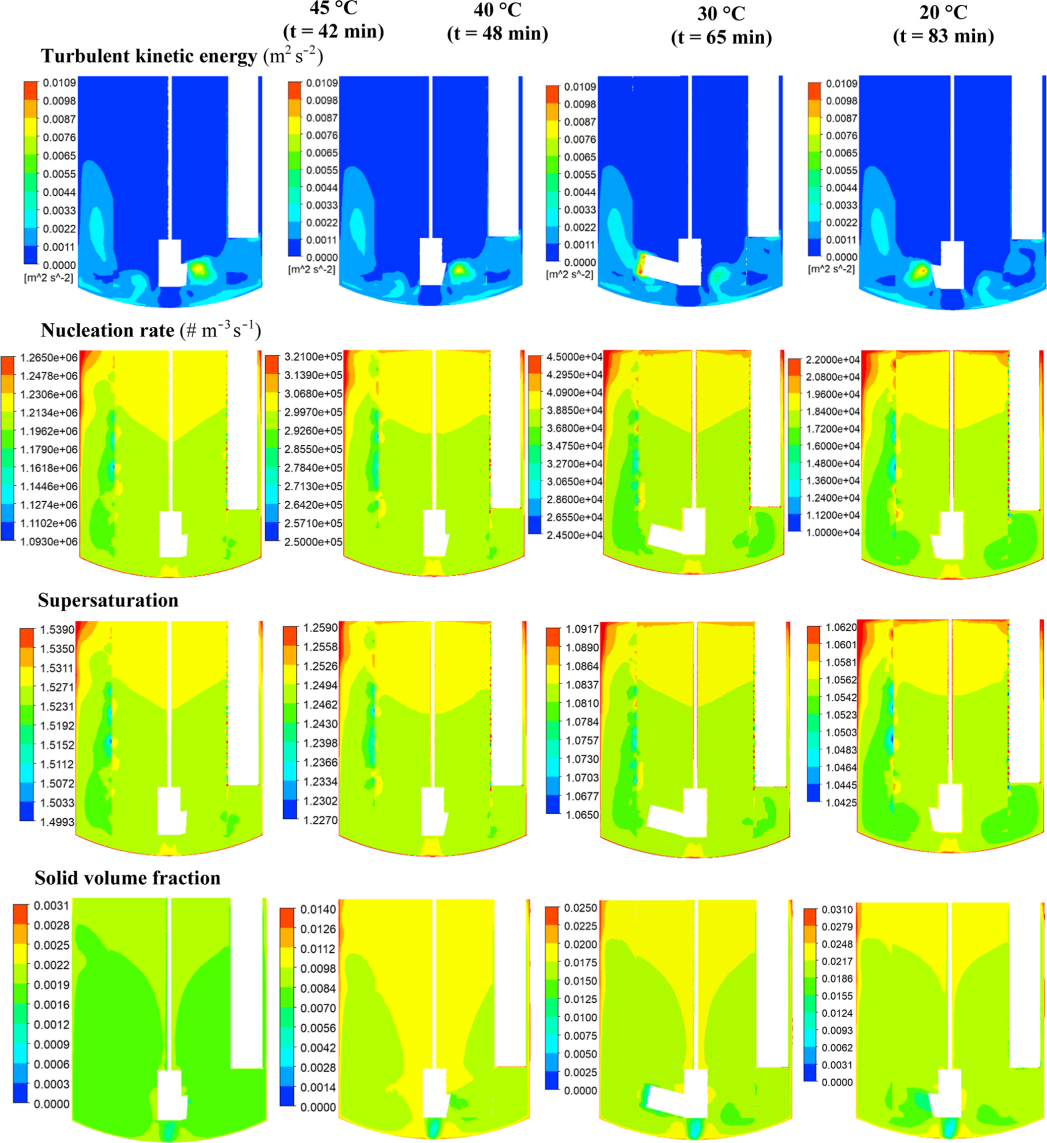
Predicted distributions of crystallization process parameters on the 0–180° vertical plane at 45 °C, 40 °C, 30 °C, and 20 °C for 150 rpm impeller speed: (a) turbulent kinetic energy, (b) nucleation rate, (c) supersaturation, and (d) solid volume fraction.

As can be seen in Figures 8 and 9, the solid volume fraction increases with the progress of the crystallization process, and at its end (at 20 °C), it becomes close in magnitude for both impeller speeds, showing larger fractions ranging between 0.022 and 0.031 in the upper region of the crystallizer, in line with the lower levels of turbulence. Fewer crystals are present in the lower region of the crystallizer with solid volume fractions between 0.012 and 0.022 where high level of turbulence exists, particularly at 150 rpm. It should be noted that at 20 °C, the supersaturation is very low, and is much more uniformly distributed for both the impeller speeds, suggesting that further growth of crystals is not significant and the solid concentration distribution patterns are largely determined by the bulk liquid flow and turbulent dispersion.

Although well-mixed mechanisti models are commonly used in crystallization process development due to their simplicity and computational efficiency, their application can be limiting when spatial effects are present. In our simulations of a batch crystallization process, it has been observed, e.g., 3D spatial and temporal variations in supersaturation within the crystallizer and that these varied, in turn, with solution agitation. These spatial differences also affect local nucleation rates, as even slight increases in supersaturation can lead to noticeably higher nucleation activity. For example, at 150 rpm (Figure 9), close to the walls or at the upper section of the vessel, although within the same order of magnitude, the nucleation rates at 30 °C are around 4 × 10^4^ #/m^3^ s compared to those at the center and lower section of the vessel which are around 3 × 10^4^ #/m^3^ s. This relative difference (∼33%) can be expected to significantly influence the CSD within the crystallization process. Even small variations of supersaturation within the crystallizer can noticeably change the nucleation rate, affecting how many primary nuclei are formed and consequently the development of the subsequent growth phase. Such variabilities can lead to measurable shifts in the CSD, particularly mindful that nucleation and growth are closely coupled and that kinetic parameters can exhibit high sensitivity.

This effect becomes more evident at an impeller speed of 100 rpm (Figure 8), where nucleation rates can differ by as much as 40% (7 × 10^4^ #/m^3^ s in the upper region vs 5 × 10^4^ #/m^3^ s in the central region of the vessel). Such heterogeneities obviously challenge the underlying assumption of homogeneous conditions within well-mixed models and may lead, in turn, to discrepancies in the prediction of nucleation behavior and the resultant CSD. Hence, while these “well-mixed” models can still provide useful insights during early R&D stages—such as for initial screening or rough estimation of kinetic parameters—they should perhaps be used with some caution through a keen awareness of their limitations. The results of this study strongly indicate the added value afforded by utilizing the complementary strengths of these two approaches, i.e., by combining mechanistic modeling with the higher fidelity CFD-based spatially resolved simulation approaches to improve accuracy and reliability, particularly when moving toward process optimization and scale-up.

### 4.4 Validation of Predicted CSD

Figure 10 shows a comparison between the predicted and measured^44^ CSDs averaged over the volume of the crystallizer at 20 °C for the impeller speed of 100 rpm (Run 1) and 150 rpm (Run 3). It also illustrates the effect of agitation rate on the CSD. Analysis of the measured CSDs reveal that the peak solid volume fraction increases and that the width of the distribution becomes narrower, shifting toward the smaller crystal sizes, as the agitation rate increases. As can be seen, the predicted CSDs in general follow the measured trends reasonably well, particularly at 100 rpm with both the predicted and measured distribution curves being centered on a very similar crystal size of 250 μm and a maximum volume fraction of 34%. However, the concentration of larger crystals (< 450 μm) is somewhat overpredicted. The predictions in line with the experimental data also reveal that the CSD curve shifts toward the smaller particles sizes at the higher impeller speed.

**Figure 10:**
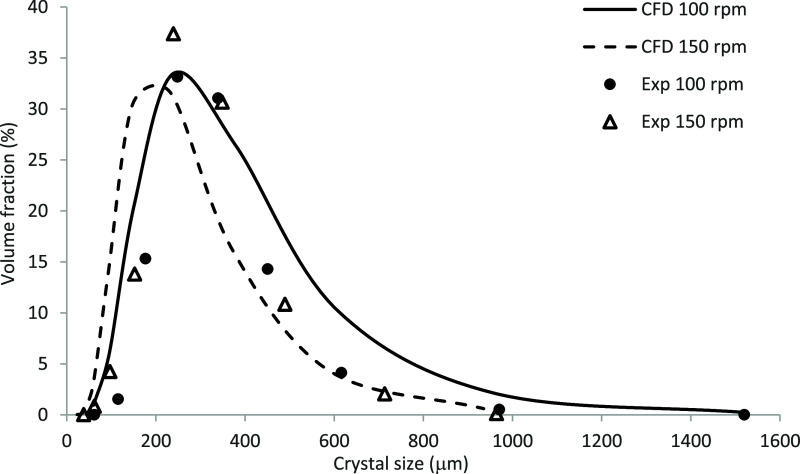
Comparison between the predicted and measured^44^ final CSDs at 20 °C for the impeller speeds of 100 and 150 rpm.

In the simulations, the effect of secondary nucleation on the overall crystallization process has been neglected, which can result in the prediction of larger crystals due to the availability of supersaturation for crystal growth in the absence of secondary nucleation. It should also be noted that the power-law growth model used in the simulations does not really account for the effect of temperature on crystal growth through the rate constant of eq 22. Fu et al.^29^ have observed that, in cooling crystallization, as the impeller speed increases, the average temperature in the crystallizer decreases, causing a decrease in growth rate regardless of the level of supersaturation. In that study, the growth rate model accounted for the effect of temperature on crystal growth via its incorporation of a temperature-dependent Arrhenius rate constant term.

### 4.5 Comparison with the Well-Mixed Case

Simulations were also carried out using a mechanistic model based on the well-mixed assumption using gPROMS software.^67^ As illustrated in Figure 11, larger discrepancies between the predictions and experimental results are observed when simulations are performed under the assumption of perfect mixing. In these simulations, the same expressions for nucleation and growth as those implemented in CFD-PBM within ANSYS Fluent were applied, ensuring a consistent comparison. In this case, inhomogeneous mixing and its influence on supersaturation and crystallization kinetics are neglected. Consequently, the predicted CSD is narrower, with most of the particle volume fraction concentrated between 100 and 400 μm. In contrast, predictions that account for mixing effects result in a broader distribution with a significant portion of the particle volume fraction shifted to sizes larger than 400 μm. As discussed previously, this difference can be explained by the impact of hydrodynamics on local supersaturation. Imperfect mixing creates spatial variations in supersaturation, which, in turn, drive different nucleation and growth rates within different mixing zones in the crystallizer. Regions of higher supersaturation promote nucleation of smaller particles, whereas areas of lower supersaturation, in contrast, favor the growth of larger crystals. As a result, the CSD predicted when mixing effects are accounted for is broader and shifted toward larger sizes. In contrast, the perfect mixing assumption neglects these spatial gradients, leading to an unrealistically narrow distribution skewed toward intermediate particle sizes.

**Figure 11:**
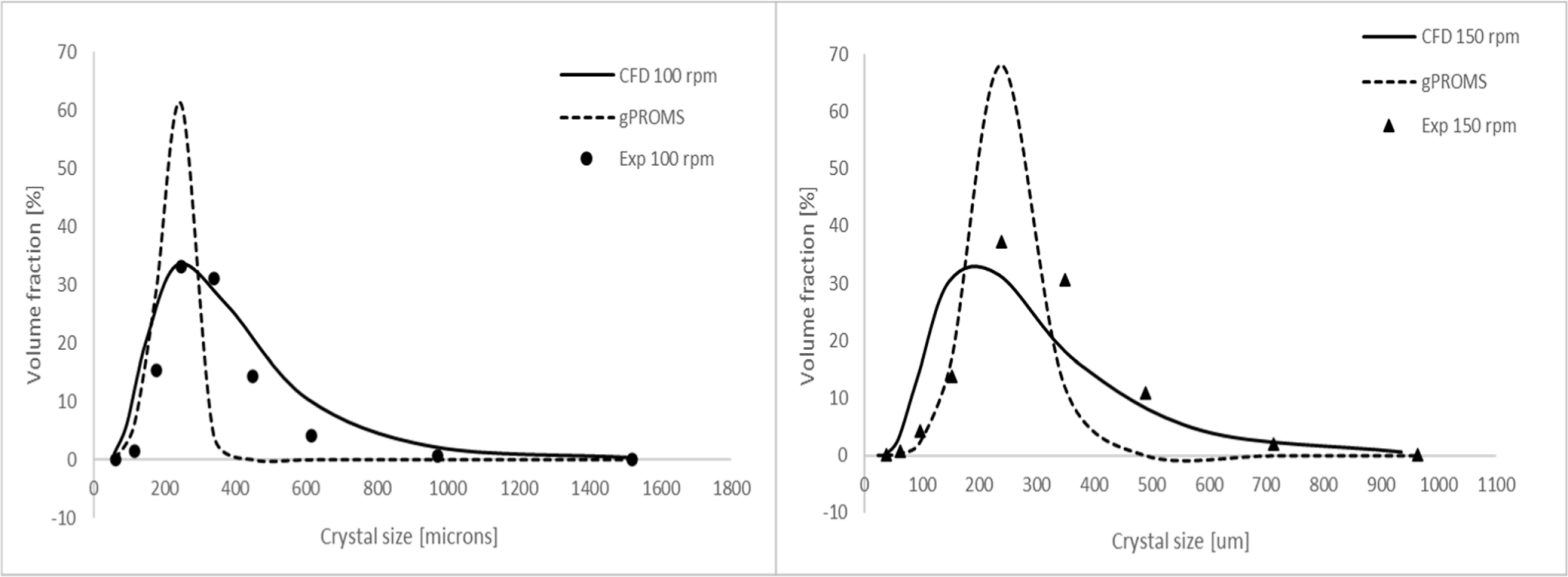
Comparison of the predicted final CSDs at 20 °C using CFD and a fully mixed mechanistic model (gPROMS^67^) with measurements^44^ for the impeller speeds of (a) 100 and (b) 150 rpm.

### 4.6 Effect of Crystal Growth Kinetics on the Predicted CSD

Figure 12 compares the CFD-PBM-predicted CSDs obtained using the crystal growth kinetic parameters of Tai and Shei^49^ and Penchev^50^ with measurements for 100 rpm (Runs 1 and 2) and 150 rpm (Runs 3 and 4). Overall, a wider CSD is predicted by the kinetic parameters determined from the data of Penchev^50^ for both impeller speeds. However, there is a clear trend that with these kinetic parameters, crystal sizes within the lower range are more accurately predicted compared with those predicted by the kinetic parameters of Tai and Shei.^49^

**Figure 12:**
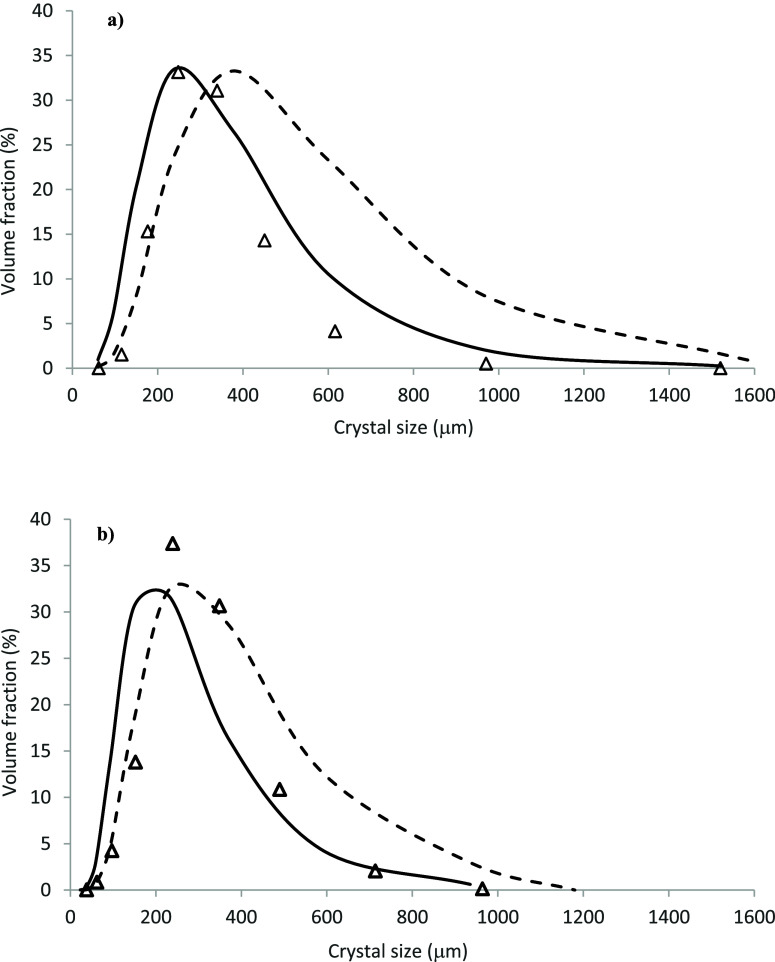
Comparison between the predicted final CSDs at 20 °C using the crystal growth kinetics parameters of Tai and Shei^49^ and Penchev^50^ and the experimental data Liang^44^ for the impeller speed of (a) 100 rpm and (b) 150 rpm.

Different predictive performances of these two sets of growth kinetic parameters can be attributed to the differences in the cooling rate, impeller speed, and crystallizer size used in the original experiments from which these parameters were determined. Tai and Shei^49^ performed crash cooling experiments at an unspecified cooling rate (although crash cooling is usually carried out at a very high cooling rate) in a 6 L crystallizer with an agitation rate of 600 rpm. This environment would be expected to produce high levels of turbulence and shear rates, which would favor secondary nucleation and enhanced mixing, leading to a smaller crystal size range. In contrast, the experiments carried out by Penchev^50^ were at a larger scale, i.e., using a 20 L crystallizer and using controlled slow cooling with relatively lower rates of temperature decrease (0.1 and 0.2 °C/min) and agitation (100 rpm) rates. In the former case, this led to a rate constant 1 order of magnitude greater than that in the latter case (see Table 3).

The poorer performance of the crystal growth model derived using the data of Penchev^50^ for the larger crystal size range may also be attributed to the difference between the conditions under which the crystal growth measurements were carried out by Penchev^50^ and CSDs were measured by Liang.^44^ The cooling rates used by Penchev^50^ (0.1 and 0.2 °C/min) were significantly lower than that used by Liang^44^ (0.6 °C/min); this would be expected to lead to higher levels of supersaturation in the latter case for which nucleation would be expected to dominate over crystal growth overall resulting in smaller crystal sizes.

## 5 Concluding Remarks

Batch cooling crystallization of α-LGA in an aqueous solution at kilo-scale size was simulated in a single baffled crystallizer agitated with a RCI by coupling an Eulerian–Eulerian multiphase CFD with a 1D-PBE. This integrated modeling approach provided a way of assessing the effect of the spatial and temporal distributions of relevant process parameters, such as supersaturation, temperature, and turbulent kinetic energy, on the nucleation and crystal growth rates, solid volume fractions, and the final product CSD under different operating conditions.

The predicted CSDs using this comprehensive modeling approach were found to be very close to those measured in a kilo-scale crystallizer, indicating its suitability for reliable simulations of industry-relevant batch cooling crystallizers in which the hydrodynamics is strongly influenced by the asymmetric configuration of the vessel. The simulation results for different experimental cases provided a detailed insight into the interactions between the hydrodynamics/mixing and the crystallization rates, resulting in the product CSDs. The simulations for two different impeller speeds revealed that at the higher speed, the CSD curve shifted toward the smaller particle sizes. This effect can be explained by the fact that when the agitation rate is higher, a greater level of turbulence and higher supersaturation at early stages of the process are achieved, enhancing nucleation over crystal growth.

PBM simulations performed under the assumption of well-mixed conditions using gPROMS^67^ with the same rate expressions for nucleation and growth as in the coupled CFD-PBM simulations reveal narrower CSDs, hence larger discrepancies between the predictions and experimental results when compared to the CFD-PBM approach. Imperfect mixing creates spatial variations in supersaturation, in turn, driving different nucleation and growth rates within different mixing zones in the crystallizer, hence resulting in the areas of higher supersaturation promoting nucleation of smaller particles, and the regions of lower supersaturation, in contrast, favor the growth of larger crystals. Therefore, the predicted CSD is broader and shifted toward larger sizes. In contrast, the well-mixed assumption neglects these spatial vatiations, leading to an unrealistically narrow distribution skewed toward intermediate particle sizes.

It is important to note that the accuracy of the predicted CSD is highly dependent on the nucleation and growth rate models (and their associated parameters) used in the PBE. The values of model parameters can depend on the crystallization conditions, such as the cooling rate and agitation rate, as well as on the crystallizer scale size. Reliable crystallization kinetics for a given solute–solvent system relevant to the crystallization conditions may not be readily available in the literature. It is therefore highly desirable to measure nucleation and growth kinetics under conditions for which simulations are being performed. In this study, two sets of kinetic parameters for the power-law crystal growth model were used. The growth kinetics of Penchev^50^ provided a better predictive performance for the smaller crystal size ranges at both impeller speeds but overpredicted the size of the crystals in the higher range. This may be attributed to the lower cooling rates used to determine the growth model parameters than those used in the crystallization experiments by Liang,^44^ as in the latter case nucleation was observed to be dominant.

The modeling methodology presented in this work is part of the development of a holistic approach for the digital design and scale-up of crystallization processes. This enables identification of the operating parameters that have the strongest influence on hydrodynamics and its effect on the crystallization process as a function of crystallizer geometry and size. In addition, drilling down into a vast amount of detailed encompassed within CFD simulation data can provide a basis for the development of the best strategy to construct a computationally expedient multizonal crystallization modeling approach with acceptable quality of predictions, as compared with the fully coupled CFD-PBM method, in order to accelerate crystallization process design and scale-up.

## Nomenclature

*C*: clearance between impeller and vessel bottom [mm]
*C*_D_: drag coefficient [−]
*C*: solute concentration [mol mol^–1^]
*C**: solute concentration at equilibrium [mol mol^–1^]
*c*_*p*_: heat capacity at constant pressure J kg^–1^ K^–1^
*D*: vessel diameter [mm]
*D*_m_: molecular diffusion coefficient [m^2^ s^–1^]
*D*_*t*_: turbulent diffusivity [m^2^ s^–1^]
*d*: impeller diameter [mm, m]
*d*_s_: particle diameter [μm, mm]
*F⃗*: external body force per unit volume N m^–3^
*G*: crystal growth rate m s^–1^
*g*: order of crystal growth rate [-]
*g⃗*: gravitational acceleration [m s^–2^]
*H*: height of liquid in the vessel [mm]
*h*_*q*_: specific enthalpy of the *q* ^th^ phase [J kg^–1^]
*J*: nucleation rate [# m^–3^ s^–1^]
*K*_sl_: interphase momentum exchange coefficient [kg m^–3^ s^–1^]
*k*: turbulent kinetic energy [m^2^ s^–2^]
*k*_N_: nucleation rate constant [# m^–3^ s^–1^]
*k*_G_: growth rate constant [m s^–1^]
*k*_*t*_: turbulent thermal conductivity W m^–1^ K^–1^
*N*: stirrer speed [rpm]
*N*_*i*_: Number of particles of size *i* [-]
*n*: order of nucleation rate [-]
*ṅ*_0_: nucleation rate [# m^–3^ s^–1^]
Pr_t_: turbulent Prandtl number [−]
*p*: pressure [N m^–2^]
*Q*_*pq*_: volumetric rate of convective heat transfer between phases [W m^–3^]
${\vec{q}}_{q}$: conductive heat flux W m^–2^
${\vec{q}}_{q}^{t}$: turbulent heat flux W m^–2^
*u⃗*: velocity vector [m s^–1^]
*u*_s_^′^: fluctuating velocity of solid [m s^–1^]
*V*_p_: volume of particle [m^3^]
Re: impeller Reynolds number [−]
Re_s_: particle Reynolds number [-]
*R*_*ij*_: Reynolds stresses [m^2^ s^–2^]
*S*: supersaturation [−]
*S*_LGA_: rate of consumption of LGA kg m^–3^ s^–1^
Sc_t_: turbulent Schmidt number [−]
*T*: temperature [°C, K]
*t*: time [s]
*V*_*i*_: volume of particles of size *i* [m^3^]
*Y*_LGA_: mass fraction of LGA in solution [-]

## Greek letters

α: phase volume fraction [−]
ε: turbulent kinetic energy dissipation rate [m^2^ s^–3^]
μ: dynamic viscosity [kg m^–1^ s^–1^]
*μ*_t_: turbulent viscosity [kg m^–1^ s^–1^]
λ: bulk viscosity [kg m^–1^ s^–1^]
ρ: density [kg m^–3^]
σ: relative supersaturation [-]
τ: stress–strain tensor [N m^–2^]
*Θ*_s_: granular temperature of solids [m^2^ s^–1^]

## Subscripts

l: liquid phase
m: solid-liquid mixture
s: solid phase

## Abbreviations

CFD: computational fluid dynamics
CSD: crystal size distribution
PBE: population balance equation
PBM: population balance model
RANS: Reynolds-averaged Navier–Stokes
RCI: retreat curve impeller
RST: Reynolds stress transport
SST: shear stress transport
USS: ultrasonic spectroscopy
